# A formal model of neighborhood representation and applications in urban building aggregation supported by Delaunay triangulation

**DOI:** 10.1371/journal.pone.0218877

**Published:** 2019-07-15

**Authors:** Tinghua Ai, Hongmei Yin, Yilang Shen, Min Yang, Lu Wang

**Affiliations:** School of Resource and Environment Sciences, Wuhan University, Wuhan, China; Shenzhen University, CHINA

## Abstract

Neighborhood relationship plays an important role in spatial analysis, map generalization, co-location data mining and other applications. From the perspective of computation, the formal model of neighborhood representation is a challenging question. This study presents a formal spatial data model for representing the planar spatial field with the support of Delaunay triangulation. Based on the three geometric elements in a triangle of the vertex, edge, and triangle area, the constructed data model describes the spatial objects of a point, line, and region respectively, as well as the neighborhood relationships among them. Three types of operators based on the model are formally defined, expanding, compressing and skeletonizing. For practical applications, three complex operators are extended by continuous and conditional operation. Through the application example of urban building generalization, this study illustrates the analysis of a neighborhood relationship and the detection of spatial conflicts, which is a crucial pre-process during map generalization. With the support of the proposed formal model of neighborhood representation, the generalization method uses the three basic operations of grouping, displacement and aggregation to perform decision making and detailed operation. The generalized result can retain the balance of built-up area better than that of other similar building generalization methods.

## 1. Introduction

Objects and phenomena in reality are represented as spatial entities and spatial relationships in a geographic information systems (GIS) conceptual world and are organized in spatial data models. Two spatial data models are commonly used: the object-oriented and field-oriented models. The object-oriented model emphasizes the integrity of entities in which the identified unit corresponds with the object in the real world, but it lacks an effective strategy for representing the inter-association or spatial relations, thus resulting in the requirement for complex vector computations. The field-oriented model focuses on the spatial correlation and continuity of the objects throughout the entire representing space. The surface is tessellated into sets of units with the attribute description of each unit, and the relations between the entities can be obtained through set-based map algebra computation, such as the expanding of the raster. With respect to which is better in spatial representation, the corresponding argument between these two models is similar to that regarding waves and particles in the physics domain in the 20^th^ century [1]. In the case of the field model, it is relatively easier to compute the spatial relationship and to conduct the spatial association processes. The map algebra method based on the raster data structure gives the field model advantage. Spatial associations, such as the neighborhood relationship, play an important role in spatial analysis, map generalization, co-location detection, and other applications. In map generalization, for example, the analysis of neighborhood relationships and detection of spatial conflicts usually comprises a significant pre-process. As the generalization attempts made to handle the spatial transformation from a large space to a small space, the spatial conflict detection within some neighboring areas is an important process. The neighborhood processing in the field model depends on the form of the data structure. For the regular data structure, such as the grid structure based on rectangles, equilateral triangle, and regular hexagons, we often use buffer operations to find the neighboring object within a certain distance. For an irregular data structure, such as the triangulated irregular network (TIN) structure, we can use the connection based on the Delaunay triangulation to find the neighboring objects. In this study, we attempt to explore the latter option by constructing a formal neighborhood model using the Delaunay triangulation and applying this in urban building generalization. In contrast to the application of TIN in the representation of the terrain surface in digital elevation model (DEM) studies, these models are applied to a planar condition with a focus on the neighborhood relation between buildings and, furthermore, on identifying spatial conflicts. However, these applications aim at different situations presenting concrete models associated with Delaunay triangulation without a unified formal model to represent neighborhood relationship. From the perspective of computation, the formal model is significant for describing the logical computation process. The formal definition and operators can be used to represent the neighborhood question in a normal and universal manner to support the algorithm design. In this study, we attempt to establish a formal model to represent a neighborhood relation based on the Delaunay triangulation to formally define spatial objects and present neighborhood-related operators.

We also attempt to apply the new model to the automatic generalization of an urban building cluster, which is associated with the neighborhood analysis. Automatic generalization of building clusters comprises different types of operations and analyses. First, it is necessary to group the buildings using different rules such as the Gestalt nature cognition or recognition of a distribution pattern. The displacement step is then used to identify how far and in which direction the objects should move. Third, the geometrical simplification and aggregation is conducted to abstract the building shape. The three steps require a model related to the neighborhood analysis to derive such descriptive indices for the distribution pattern, distribution density, adjacency direction, adjacency distance, etc.

In building data generalization, the operation can be classified into two types according to the object type, that is individual building generalization and cluster building generalization. Several works have developed algorithms and methods for independent building simplification [2–18]. Regnauld and Edwardes [2] proposed three typical operations for the simplification of buildings from the perspective of the readable view: squaring, detail removal, and local enlargement. By separating a building into some hierarchical rectangle elements, Guo and Ai [4] presented a method to simplify the building polygon based on an idea of divide-and-conquer. By treating the four continuous adjacent points as an overall unit, Xu et al. [7] proposed an approach for building simplification based on the improvement of local simplification algorithms. In this method, the bend structures can be analyzed and the short edges can be removed. Sester [13] proposed an approach of building simplification comprising the use of a set of rules and adjusted the simplified building optimally based on the theory of least squares adjustment. In this method, the short edges can be eliminated using an optimization solution. In terms of artificial intelligence, Cheng et al. [17] presented a model of back-propagation neural network model for learning cartographers’ knowledge for the realization of building simplification. However, these studies usually only took into consideration the geometric features and ignored the neighborhood relationships of the building during the generalization.

As compared with the individual building simplification, the building cluster generalization is more complex and requires the consideration of a greater number of aspects, such as the group pattern detection, context analysis, and spatial conflict judgment. An efficient model is thus required for the neighborhood analysis. Regnauld [19] presented an approach for classifying building groups using the multiple spanning tree model in graph theory. Li et al. [20] proposed an approach for group building cluster that combined the Gestalt theory and urban morphology. Chaudhry and Mackaness [21] combined the buffer of building objects and the derivation of the single building object surrounding clusters for the generalization of city boundaries. Shen [22] et al. applied the superpixel segmentation technologies for building aggregation. Proximity analysis plays a very important role in building generalization because displacement conflicts or topology errors may be produced during the generalization. Thus, we attempt to apply the proposed neighborhood representation model to the automatic generalization of urban building clusters. An outstanding property exists in our neighborhood analysis model, in that, it can simultaneously support several operations including grouping detection, displacement, and aggregation. The minimum spanning tree (MST) model in Regnauld’s method [19] and the urban morphology model in [20] can be used to conduct only one operation aggregation. The difference between our method and the others is the change in the built-up area after the map generalization. Our proposed method is effective in balancing the built-up area as the displacement prevents the gap area from being absorbed into built-up area.

This paper is organized as follows: the neighborhood representation model of the spatial field is discussed in Section 2. Section 3 presents the expanding and compressing operation based on the proposed formal triangulation data model (FTDM). A progressive algorithm of building cluster aggregation based on FTDM is presented in Section 4 with experiment illustrations, and some future improvements are discussed in the conclusion in Section 5.

## 2. Neighborhood representation model based on Delaunay triangulation

**Fig 1:**
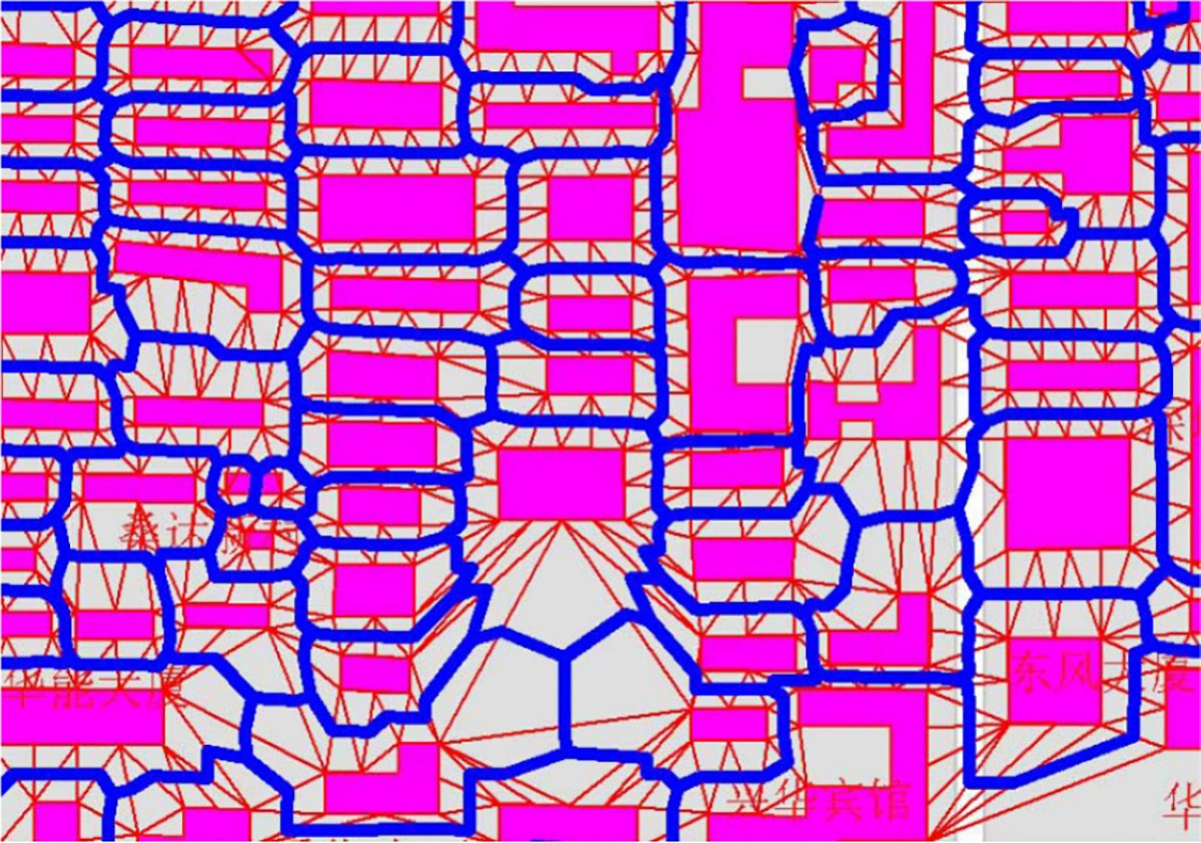
Detecting the neighboring region among building cluster using Delaunay triangulation.

In the field-oriented model, the TIN structure covers the whole region without overlaps or gaps between the tessellated triangles. The relations among spatial entities are represented by the connection of triangles. As a special geometrical construction to support the TIN establishment, the Delaunay triangulation has the characteristics of “circumcircle rule” and “maximum rule of the smallest angle,” and these properties make it an efficient model for representing the spatial neighborhood relationship [23, 24]. In the domain of map generalization, the Delaunay triangulation and its dual Voronoi diagram are widely used in the detection of conflict and neighbor relationships between objects, thus generating various data models aimed at different purposes. For example, Jones and Ware [25, 26] built the simplicial data structure (SDS) model for searching for area cluster targets and merging the adjacent objectives; the enhanced formal data structure (EFDS) model developed by Peng [27] was an improved model of the formal data structure (FDS) model developed by Molenaar, is which focused on the extraction of a “safe area” and “non-safe area” in the target generation space. Other works in which the Delaunay triangulation was applied to map generalization include the building pattern recognition before generalization [28], the displacement of crowded building and conflicted point symbol [29, 30, 31], line simplification [32–35], polygon decomposition during land-use data generalization [36], and watershed area computation in hydrographic network generalization [37]. These properties make the Delaunay triangulation an efficient tool in GIS modelling applications. One such typical application is the representation of the terrain surface by TIN model. However, the aforementioned property of the Delaunay triangulation also makes it an efficient tool for neighborhood representation in a planar space, as shown in Fig 1. Through the triangle connection, the neighborhood relation among the spatial objects in the two-dimensional space can be detected. In this section, we attempt to build a formal model of neighborhood representation using the Delaunay triangulation.

### 2.1. Formal definitions

The spatial objects including points, lines, and polygons must be embedded in the Delaunay triangulation and then through the triangle edges to detect the neighborhood relation. We build a model to involve all the spatial objects to be studied and use the vertices of linear objects and area objects be a point set for the triangulation construction.

We first determine the geographical extent of the spatial representation. We then add three points *Q*_1_, *Q*_2_, and *Q*_3_ to ensure that all the spatial objects are within the triangle identified by the three added points. The Euclidean space containing Δ*Q*_1_*Q*_2_*Q*_3_ is denoted as Δ. We use the planar points to construct the constrained Delaunay triangulation, which is expressed as *W*⟨*V*,*E*,*T*⟩, where *V* = {*v*_1_,*v*_2_,⋯,*v*_*m*_} is a non-empty point set, *E* = {*e*_1_,*e*_2_,⋯,*e*_*n*_} is a non-empty edge set, and *T* = {*t*_1_,*t*_2_,⋯,*t*_*n*_} is a non-empty triangle set.

Obviously, *W* is a graph *G*⟨*V*, *E*⟩ with *V* as the vertex set and *E* as the edge set. The edge *e*_*i*_∈*E*, is represented by a point pair comprising the points of set *V* as *e*_*i*_(*v*_*i*0_,*v*_*i*1_). The triangle *t*_*i*_∈*T*, comprises the three edges of set *E* and is represented as *t*_*i*_(*e*_*i*1_,*e*_*i*2_,*e*_*i*3_). The triangle set *T* covers the entire domain space. For any *t*_*i*_,*t*_*j*_∈*T*, there is *t*_*i*_∩*t*_*j*_ = Null or DIM (*t*_*i*_∩*t*_*j*_) = 1, where the latter means that the two triangles are adjacent and the dimension of the intersect region is 1, i.e., a line. When there is a unique sharing edge between triangles *t*_*i*_ and *t*_*j*_, namely, ∃*u*,v:*e*_*iu*_ = *e*_*jv*_, triangles *t*_*i*_ and *t*_*j*_ are called adjacent.

**Fig 2:**
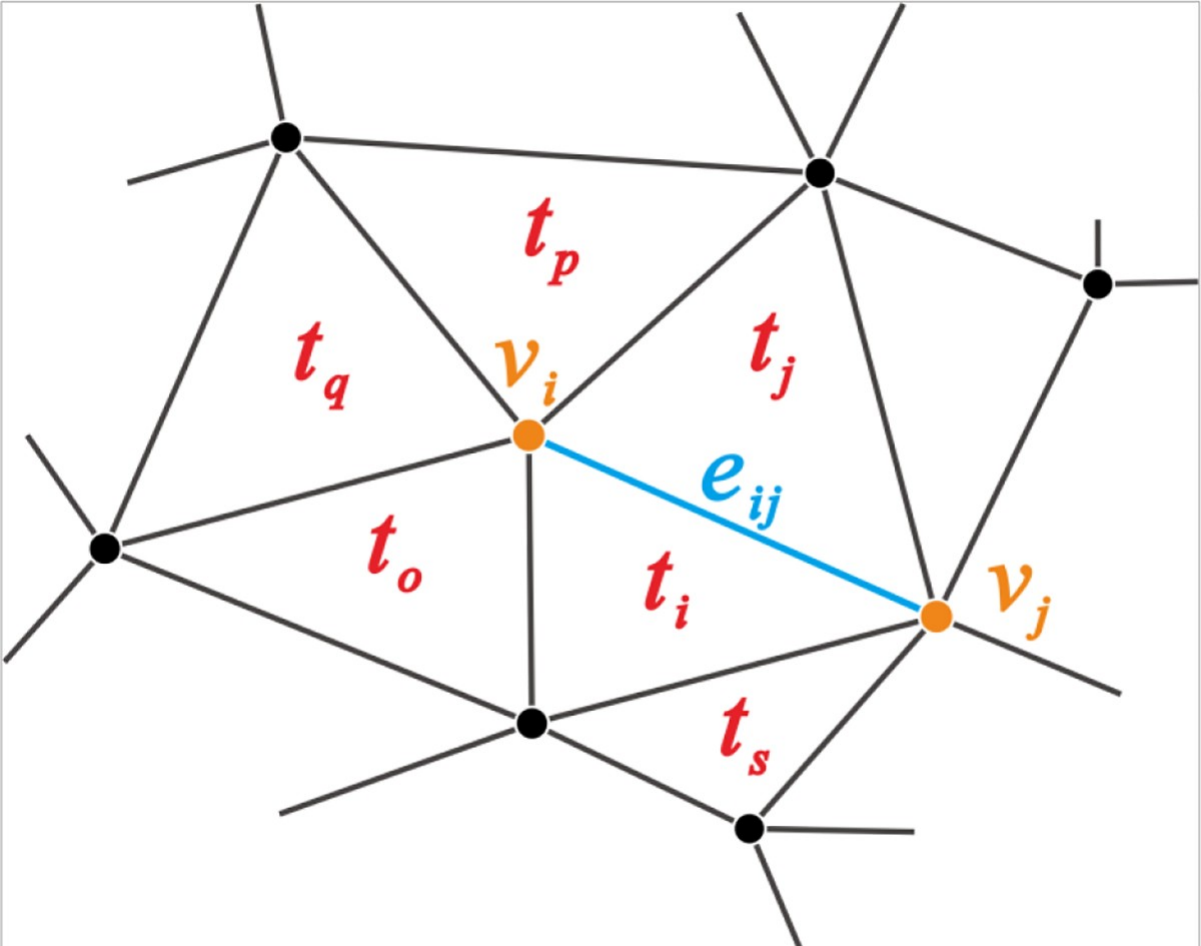
Formal definitions of the functions related to neighborhood analysis.

We define the functions related to the neighborhood analysis as follows:

*Neighbor(t_i_,e_ij_)*: *T*×*E*→*T* returns the triangle *t*_*j*_ that is adjacent to triangle *t*_*i*_ and the sharing edge is *e*_*ij*_. As shown in Fig 2, the triangle *t*_*i*_ and *t*_*j*_ has the sharing edge *e*_*ij*_.
*Neighbors*(*t*_*i*_): *T*→*T* returns the set of triangles that are adjacent to *t*_*i*_, and:
*Neighbors*(*t*_*i*_) = {*Neighbor*(*t*_*i*_, *e*_*i*1_), *Neighbor*(*t*_*i*_, *e*_*i*2_), *Neighbor*(*t*_*i*_, *e*_*i*3_)}. As shown in Fig 2, the neighboring triangles of *t*_*i*_ are *Neighbors*(*t*_*i*_) = {*t*_*j*_,*t*_*o*_,*t*_*s*_}.
*Joins*(*v*_*i*_): *V*→*T* returns the set of triangles that shares the vertex *v*_*i*_. In Fig 2, *Joins*(*v*_*i*_) = {*t*_*i*_,*t*_*j*_,*t*_*p*_,*t*_*q*_,*t*_*o*_}.
*Condition*(*c*): {logic expression *c*}→*T* returns the triangle set *T*_*c*_ that satisfies condition *c*.
*Access(t_i_,t_j_)*: *T*×*T*→{TRUE, FALSE}, when there exists one set of edges between any vertex of *t*_*i*_ and vertex of *t*_*j*_ in graph *G*⟨*V*, *E*⟩, returns TRUE or else returns FALSE. In Fig 2, *Access* (*t*_*p*_,*t*_*s*_) = *true*, as there is path connecting triangle *t*_*p*_ and *t*_*s*_.
*Begin*(*e*): *E*→*V* returns the start point *v*_*b*_ of edge *e*.
*End*(*e*): *E*→*V* returns the end point *v*_*e*_ of edge *e*.

### 2.2. Representation of spatial objects

We denote the set including all the objects within the space as *F*_Δ_, which includes all the point objects *P*_Δ_, all linear objects *L*_Δ_, and all area objects *A*_Δ_. We then have *F*_Δ_ = *P*_Δ_∪*L*_Δ_∪*A*_Δ_. We now define the triangulation model representing space objects called FTDM for short.

**Point object:** *p*∈*P*_Δ_ is defined such that if the element *v*_*i*_ belongs to *V*, then for any point *p* belonging to *W*⟨*V*,*E*,*T*⟩ there is *p*∈*V*.

**Line object:** *l*∈*L*_Δ_ comprises a series of line segments. In *W*⟨*V*,*E*,*T*⟩, these segments must belong to *E*. *l* can be defined as: the ordered subset {*e*_0_,*e*_1_,⋯,*e*_*n*_} in *E* that satisfies the condition *end*(*e*_*i*_) = *begin*(*e*_*i*+1_) for any *i*∈[0,*n*−1].

**Area object: *a*∈*A*_Δ_** can be defined as the subset {*t*_0_,*t*_1_,⋯,*t*_*m*_} that satisfies the condition Access (*t*_*i*_,*t*_*j*_) = TRUE for any *i*,*j*∈[0,*m*].

**Fig 3:**
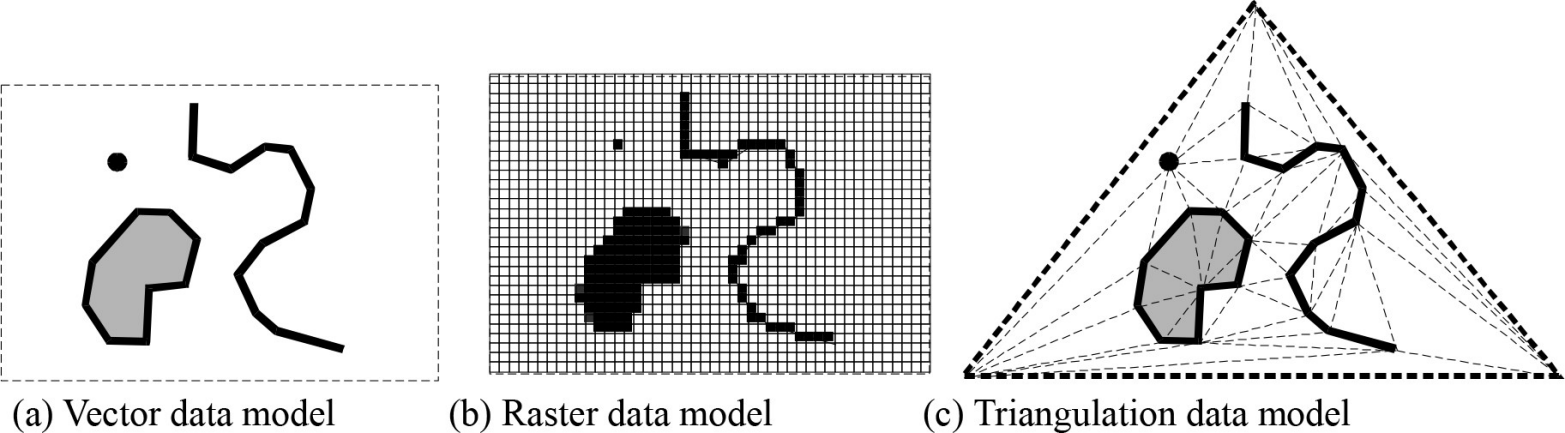
The representation of spatial objects in three data models.

Fig 3 shows that the representation of various spatial objects in the FTDM model, which is obviously different from that in the raster model, which uses a regular grid unit to represent a point, line, and region. The FTDM model comprises three elements: a point unit, boundary unit, and triangle unit, while there is only one structural unit in the raster model. Triangles in the FTDM model play two roles: the component of area object and the connection between objects. The triangles playing the connecting role can connect objects according to "visibility" between the objects and "the nearest link" based on the Delaunay triangulation, irrespective of the geometric distance between the objects. However, in the raster model, the connection between objects is usually formed by the ordinal linkage of a number of structural units. When searching for the neighboring object, the raster calculation is necessary for obtaining the results, and the four- or eight-direction expansion in the raster calculation is not only blind, but also has no neighboring connecting properties of triangulation.

## 3. Neighborhood operators based on the FTDM model

### 3.1. Simple operators

For simulating the operation of the raster model, two types of operators of FTDM are defined based on the constrained Delaunay triangulation *W*⟨*V*,*E*,*T*⟩: expanding and compressing. Firstly, we define the region of the triangle set and related concepts. Region r is defined as the subset of *T* that satisfies certain conditions, and we let *r* be the single connected region, i.e., *r* = *Condition*(*c*)⊂*T*. The boundary *b* of region *r* is represented as follows:

$$b=Boudary(r)=\{e_{i}|\exists t:t\in Condition(c)\wedge Neighbor(t,e_{i})\notin Condition(c)\}$$

The boundary defined here is the set of the edges of the triangles, without considering the situation that organized them in an orderly manner to form a closed ring.

Based on boundary *b*, we denote the outside neighbor of the boundary *b*_*out*_ and the inside neighbor of the boundary *b*_*in*_ as follows:

$$b_{in}=\{t_{i}|t_{i}\in r\wedge (\exists e:e\in b\wedge (t_{i}\in Joins(begin(e))\vee t_{i}\in Joins(End(e))))\}$$

$$b_{out}=\{t_{i}|t_{i}\notin r\wedge (\exists e:e\in b\wedge (t_{i}\in Joins(begin(e))\vee t_{i}\in Joins(End(e))))\}$$

Based on *b*_*in*_ and *b*_*out*_, we define the regional expanding operator *Expand*() and regional compressing operator *Compress*() as follows:

*Expand*(*r*):*T*→*T*, the transformation of region *r* that meets condition *c*, the return value is *r*∪*b*_*out*_.
*Compress*(*r*):*T*→*T*, the transformation of region *r* that meets condition *c*, and the return value is *r*−*b*_*in*_.

**Fig 4:**
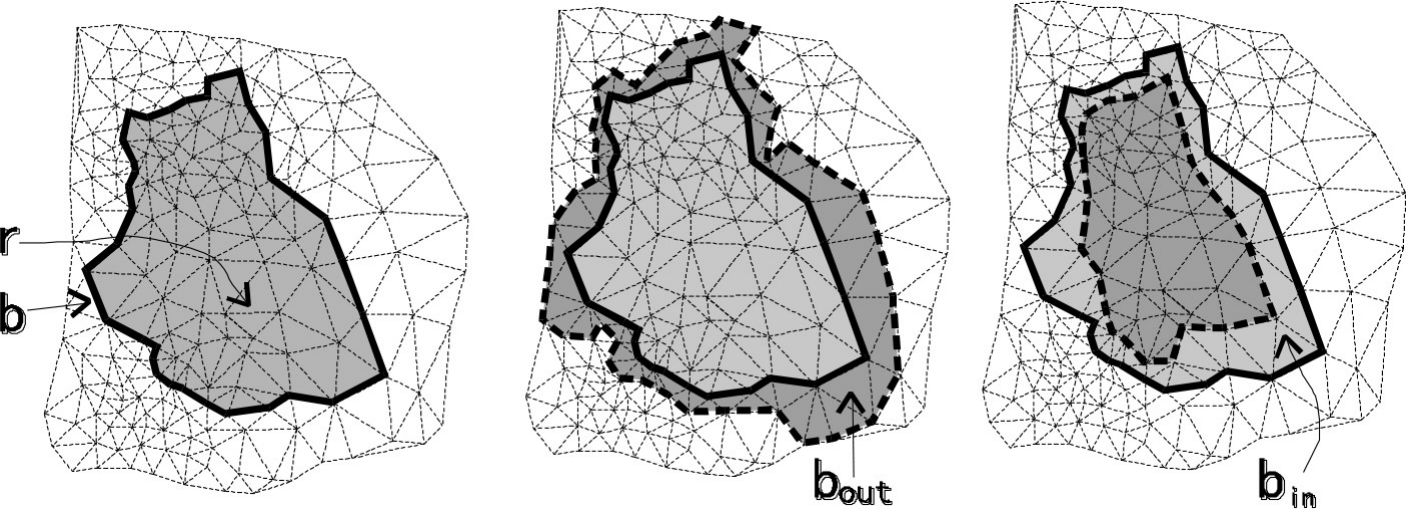
The illustrations of region expanding and compressing. ***r*** denotes the region, ***b*** the boundary, ***b_in_*** the inside neighbor, and ***b_out_*** the outside neighbor.

The transformation result of the two operators is presented in Fig 4.

### 3.2. Complex operators

The expanding and compressing operations can be applied to the region iteratively. We introduce the continuously expanding operator denoted as *Expand*^(*n*)^(*r*), and the continuously compressing operator denoted as *Compress*^(*n*)^(*r*). The recursive definition for *Expand*^(*n*)^(*r*) is

$$Expand^{\left(0\right)}\;\left(r\right)=r;$$

$$Expand^{\left(n\right)}\;\left(r\right)=Expand\;\left(Expand^{\left(n-1\right)}\;\left(r\right)\right).$$

The recursive definition for *Compress*^(*n*)^(*r*) is

$$Compress^{\left(0\right)}\;\left(r\right)=r$$

$$Compress^{\left(n\right)}\;\left(r\right)=Compress\left(Compress^{\left(n-1\right)}\;\left(r\right)\right);$$

*Compress*^(*n*)^(*r*) results in the repetitive peeling of the region, and *Expand*^(*n*)^(*r*) results in the gradual expansion of the region.

The operator *Expand*(*r*) and *Compress*(*r*) for the region *r*∈*T* is non-conditional, but for some operation in the real application, it is necessary to perform conditional filtering for the expanded triangular set *b*_*out*_ and the eliminated triangular set *b*_*in*_ of *Expand* and *Compress*, respectively. Therefore, we further define the conditional expanding operator *Expand*_*c*_(*r*) and the conditional compressing operator *Compress*_*c*_(*r*).

Let us suppose the condition is *c*_0_, then

$${Expand}_{c}(r):T\to T,return\;r\cup (b_{out}\cap Condition(c_{0}));$$

$${Compress}_{c}(r):T\to T,return\;r-(b_{in}\cap Condition(c_{0}));$$

The return value can be several non-connected subsets of triangles.

The introduction of conditional restriction in the continuous operator results in a conditional continuous operator, which is denoted as *Expand*_*c*_^(*n*)^(*r*) and *Compress*_*c*_^(*n*)^(*r*), respectively. The conditions for *Expand*_*c*_^(*n*)^(*r*) and *Compress*_*c*_^(*n*)^(*r*) can be classified as follows.

(1) Target achieved condition

In the expanding or compressing operation, when the expanded or eliminated triangle has a certain relation with the spatial target, then the operation is stopped. This relation includes when the vertex of the triangle reaches the appointed point target, the edge of the triangle is attached to any edge of the line target or area target, or the polygon is attached to any area target. Under this condition, the neighbor relationship between objects can be determined via two operations.

(2) Geometric feature condition

When the perimeter of the triangle, area of the triangle, length of the arc, or area of the closed ring clipped by the edge of the triangle on the edge of the polygon meet the threshold conditions, then the operation is stopped. The triangles in the triangulation *W*⟨*V*,*E*,*T*⟩ are merely connected based on the visibility between targets. The spatial distance should be considered when judging the contiguity of the triangles, and there should be some restrictions on the length of their edges.

(3) Spatial measurement condition

During the procedure of expanding or compressing, we consider the distance or orientation from the edge of the region, center of the region, or other reference locations as the restrictions. This is suitable for the operation on the anisotropy spatial field. Weights should be added to the expanse of the operation in different directions.

(4) Neighboring freedom condition

The neighboring expanding in a raster model comprises two cases: eight-direction and four-direction expansion. Similarly, the operation in the FTDM model has edge neighboring expanding and point neighboring expanding. In the above research, the application of function *Joins*(*v*_*i*_) is point neighboring muti-direction freedom. When the adjacent connection for triangles is restricted, the freedom is three.

From the practical judgment point of view, the above conditions can be determined using two classes. One class is based on qualitative judgment for selecting the conditional region in which the triangle touches certain types of objects or with a connection relation to certain object. The other class is based on quantitative measurement for selecting the conditional region in which the triangle area, edge length, and movement angle exceed a pre-defined tolerance, such as the area of 4 mm^2^, distance of 2 mm, and the angle of 0.5 π. In the following section 4, an example will be presented to illustrate the continuous conditional operator *Expand*_*c*_^(*n*)^(*r*) through a distance measure to determine how to expand and then finally stop.

It is worth noting that although the operators of *Expand*(*r*) and *Compress*(*r*) have opposite effects on the change in the region extending range, they do not generally satisfy the equation: *r* = *Compress*(*Exprend*(*r*)) or *r* = *Expand*(*Compress*(*r*)). This is because the restrictions in the expansion and compression procedure vary in different directions, and it thus is impossible to achieve an isotropic effect.

### 3.3. Skeletonizing operator

The use of a refinement operator in the raster model is one method for converting the raster into the vector skeleton. In the FTDM model, we can also define the skeletonizing operator *Skeleton(r)*, which is a function that converts region *r* into the graph structure related to a skeleton structure.

We first classify the triangles in the conditional region *r* = *Condition*(*c*) into three types. We define function *f*(*t*) as *Condition*(*c*)→{0,1,2,3}. The return value is the number of elements in the set of *Neighbors*(*t*)∩*Condition*(*c*), which is denoted as *f*(*t*) = ‖*Neighbors*(*t*)∩*Condition*(*c*)‖. We classify the triangles in *r* into three types: if *f*(*t*) = 1, then *t* belongs to Type I; if *f*(*t*) = 2, then *t* belongs to Type II; if *f*(*t*) = 3, then *t* belongs to Type III; and if *f*(*t*) = 0, then *t* is isolated triangle, out of consideration. To facilitate the narrative, we call the shared edge between *t* and *Neighbors*(*t*) as the neighbor edge.

We now define the regional skeletonizing operator *Skeleton*(*r*). Let us suppose that the set of all graphs is *G*_Δ_, then *Skeleton(r)*: *T*→*G*_Δ_, the return value is *G*⟨*E*’,*V*’⟩, where *E*’ and *V*’ are the generated set of edges and the set of vertices, using the skeletonizing method, respectively.

**Fig 5:**
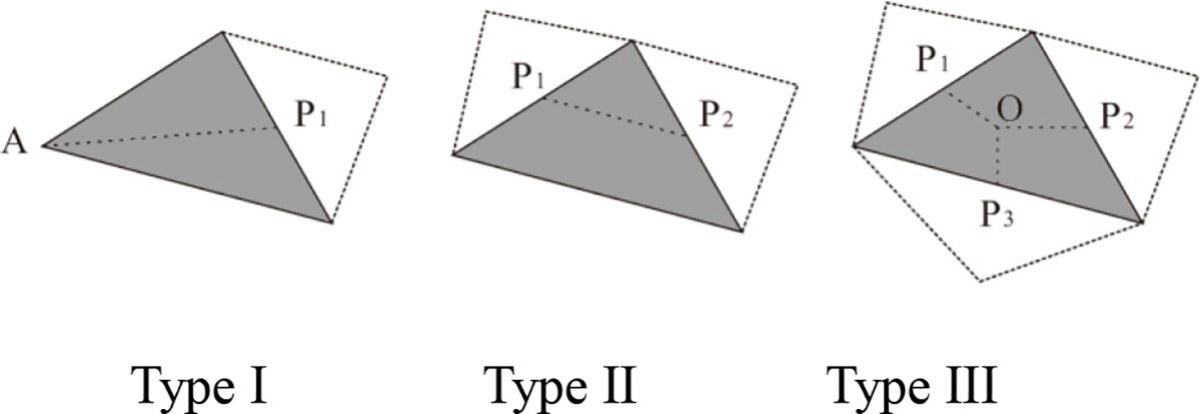
The three types of triangle and the skeleton line connection for each one.

As shown in Fig 5, for Type I triangles, we connect the mid-point of the unique neighbor edge with the corresponding vertex; for Type II triangles, we connect the mid-points of the neighbor edges; while for Type III triangles, we connect the center of gravity with the mid-points of all the edges.

$$\mathrm{Type}\;I:\;A\to P_{1}\;\mathrm{or}\;P_{1}\to A;$$

$$\mathrm{Type}\;\mathrm{II}:\;P_{1}\to P_{2}\mathrm{or}\;P_{2}\to P_{1};$$

$$\mathrm{Type}\;\mathrm{III}:\;O\to P_{i}\mathrm{or}\;P_{i}\to O,\;i=1,2,3.$$

Starting from Type I or Type III triangles, searching along the neighbor relationship across Type II triangles and ending with Type I or Type III triangles, we can obtain one edge of the graph formed by a series of points {*Q_i_*}.The construction of the graph is finished when all the Type I triangles have been searched once, and all the Type III triangles have been searched three times.

**Fig 6:**
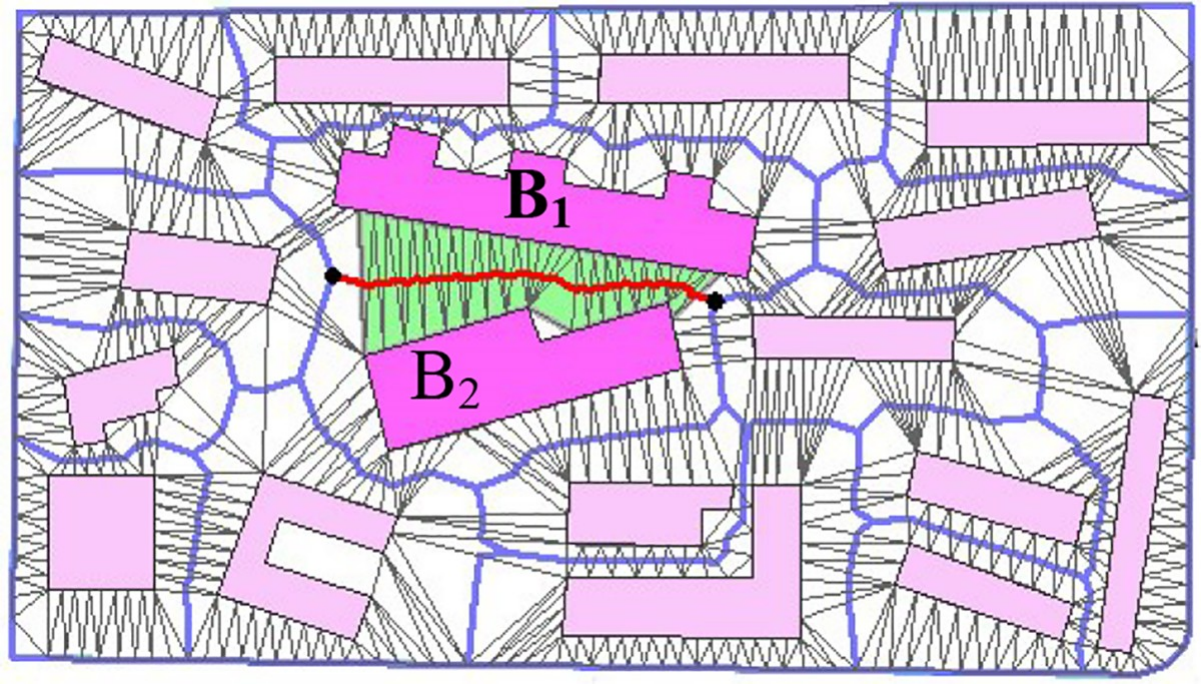
The extraction of the skeleton from the triangle region.

For the extracted graph structure, as shown in Fig 6, each edge (indicated by a red line) corresponds to a skeleton between two boundaries of two objects *B_1_* and *B_2_* on two sides. This represents the skeleton across a set of triangles (indicated in green) connects two objects (*B_1_* and *B_2_* in Fig 6).

**Fig 7:**
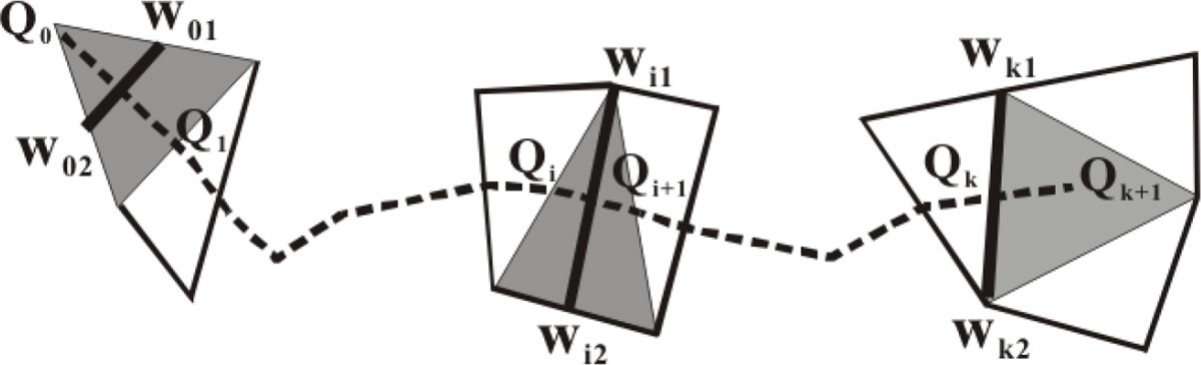
The local distance representation of W_i1_W_i2_ for three types of triangles.

We can compute the distance between two objects based on the average height of the triangles crossed by skeletons. As shown in Fig 7, we scan all the triangles across the skeleton. We compute the local distance for each triangle and then integrate the weighted distance as the average distance between two objects. For three types of triangles, the corresponding local distance *W_1_W_2_* is presented in Fig 7. The computation function of the average distance ŵ is

$$ŵ=\sum_{i=0}^{k}\frac{\left\|Q_{i}Q_{i+1}\right\|}{l}\|W_{i1}W_{i2}\|$$

where *l* is the length of the entire skeleton, and *k* is the number of triangle involved. ŵ is also called the skeleton width. This weighted distance computation based on the skeleton takes into consideration the building shape structure, spatial distribution, and other building’s influence.

**Fig 8:**
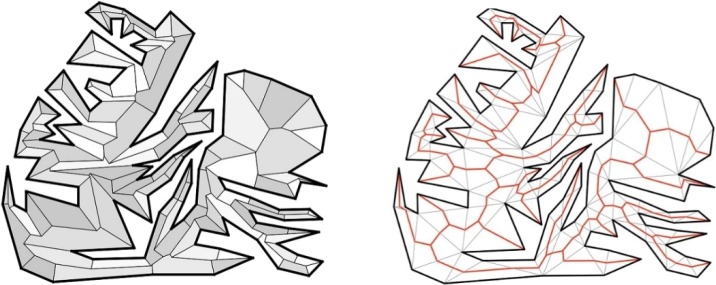
A comparison between the MAT-based skeleton (left) and Delaunay-triangulation-network-based (DTN-based) skeleton (right) using the same complex polygon data.

The skeleton operator can obtain the main central line of a region, such as a polygon. In the field of geometric shape analysis, there also exist other methods for extracting the polygon central line, for example, the medial axis transportation (MAT) based on the angular bisector operation [38], the superpixel segmentation method [39]. We use the same polygon data with a complex shape for conducting the comparison between the MAT-based method and the Delaunay-triangulation-based method. From the result of the example that is presented in Fig 8, it can be observed that the Delaunay-triangulation-based method has fewer hair segments, and the skeleton shape is smoother than that of the MAT-based method. An in-depth comparison of these two methods is outside the scope of this study, while a detailed discussion of the different methods can be referred to in [4] and [38].

**Fig 9:**
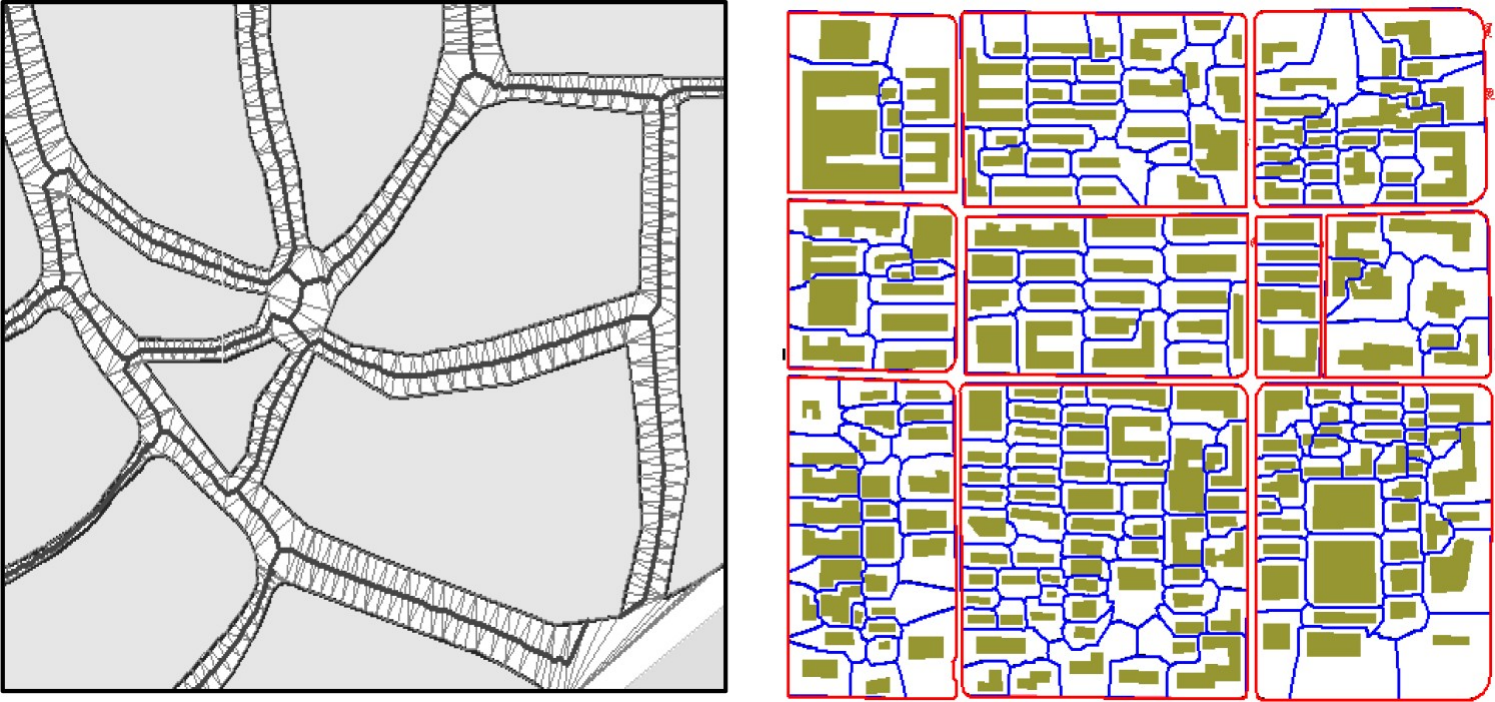
(Left) performing skeletonizing outside street blocks results in the medial street line, (Right) performing skeletonizing on building clusters within a street block.

The operator skeletonizing is performed on the region covered by the triangle set. We use different condition *c* to extract a subset of triangles, and the skeletonizing results in different graphs for representing the corresponding geometric structures. For a polygon cluster, if we extract the triangle region within the polygon, the skeletonizing will obtain the medial structure line as shown in Fig 9 (left). This process can be used to simplify the hydrographic features by collapsing narrow polygons into a line representation. If we extract the triangle region of the street roads (outside a street block), the skeletonizing provides the medial line of the street features for supporting applications such as navigation. If we extract the triangulated region outside the building polygons, the skeletonizing provides a similar Voronoi diagram to tessellate the polygon cluster, in which the neighboring polygon is separated by the connected skeleton line, as shown in Fig 9 (right).

## 4. Application of FTDM model in building cluster generalization

The building or built-up area in an urban region has special properties with respect to distribution structure, cluster pattern, and alignment shape. In spatial data scaling, the generalization of building data within street block is an attractive question requiring special strategies to consider the aforementioned properties [40]. Some methods have been developed to settle the abstraction of a building within a street block, such as the aggregation under the control of Gestalt principles and urban morphology [20], the application of an optimization method in building cluster abstraction [12], and using the MST model to group building clusters [19]. These methods can be used to conduct only one operation: either building group detection or neighbor building aggregation. The resulting issue is that the aggregated building will greatly increase the built-up area as the gap area between the neighbors has been included in the built-up region. With the objective of resolving this issue, we develop a method based on the FTDM model to perform building generalization to maintain the balance of the built-up area as well as possible. We use the FTDM model to detect the competition area and perform displacement and aggregation together to prevent the gap area from becoming absorbed into built-up area.

We define the building cluster generalization such that it includes four steps: grouping, displacement, aggregation, and simplification. The grouping step comprises the detection of the cluster pattern, which should be preserved after abstraction. The displacement operation attempts to move buildings within a group closer to avoid increasing the built-up area. The aggregation operation attempts to combine the closed building group into one block. The final simplification step is used to make the shape of the built-up area simple with orthogonal characteristics. The proposed FTDM model can be used to support the generalization of urban building clusters in several aspects. Through triangle tessellation and neighborhood analysis by triangle expansion, we can detect the building group and conflict area and further through skeletonizing operations, move closed buildings together. It is the displacement that guarantees the balance of the built-up area after the building data generalization. In this section, we present the application of the FTDM model in building data generalization and focus on three operations: grouping, displacement, and aggregation.

**Fig 10:**
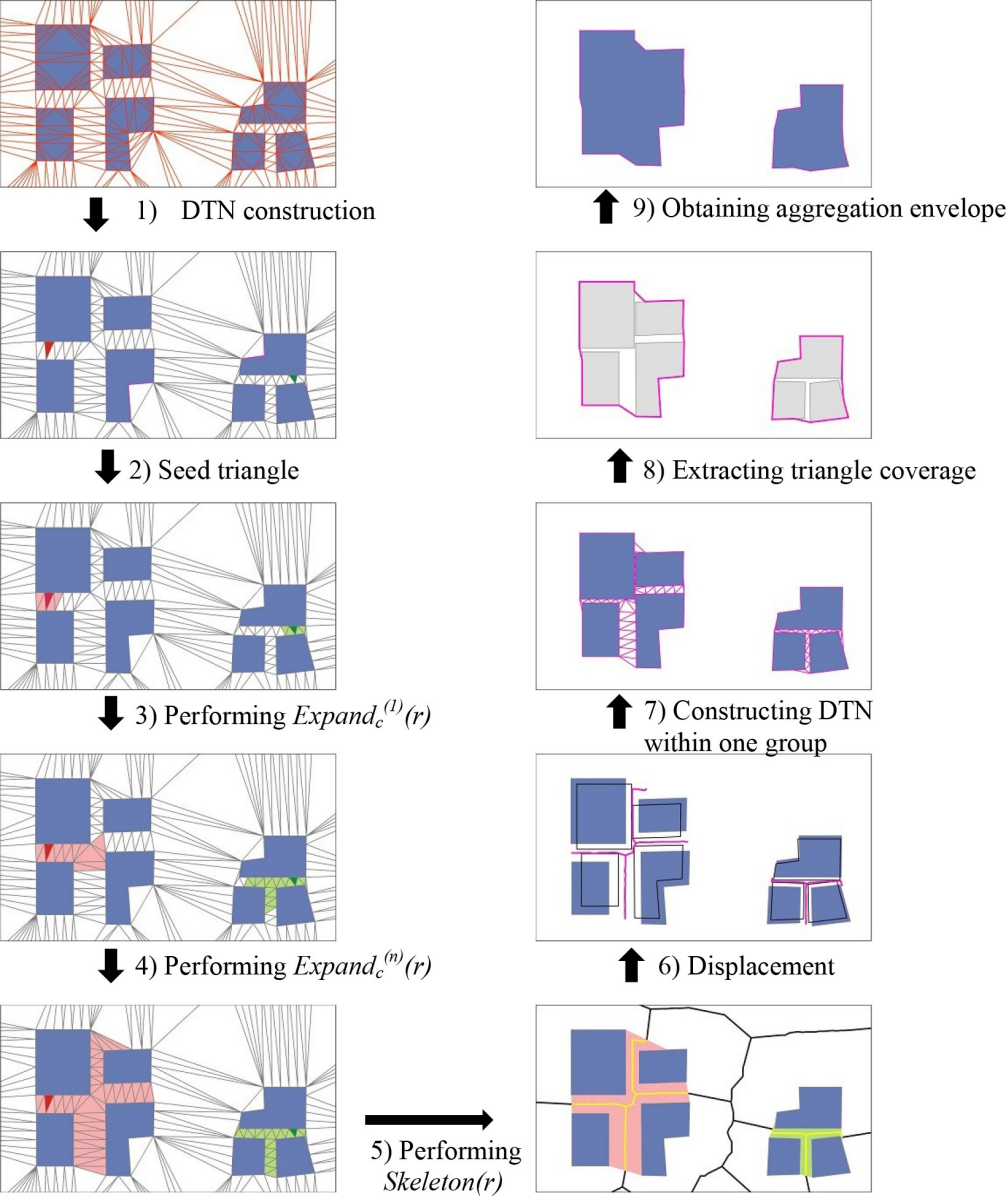
The illustration of the building aggregation using FTDM and operators *Expand(r)*, *Skeleton(r)*.

The entire process of the building data generalization is based on the FTDM model, comprises nine steps, and is illustrated in Fig 10. The first constructed triangle set covers the whole building area and obtains the tessellation as the FTDM model to support the next neighborhood analysis. We then use the formal operations *Expand(r)* and *Skeleton(r)* in the FTDM model to extract the building group and further to move the closed building together. Finally, the aggregation of the moved neighbors provides the generalization result. In the nine steps of the building generalization, the formal operator *Expand(r)* and *Skeleton(r)* in the FTDM model plays an important role.

### 4.1 Grouping and conflict detection

As shown in Fig 10 (from step 1 to 5), after the construction of the building cluster triangulation we obtain the FTDM model. For any building object, we select one triangle (indicated in red or green in Fig 10) touching it as the expansion seed. Then, we perform *Expand_c_^(n)^(r)*. This is a continuous and conditional expansion operation (see its definition and performing function in section 3). The condition here is that the triangle should be located outside the building polygon, and the length of the triangle edge between the neighboring buildings should be less than the pre-defined tolerance *d*. The value of *d* depends on the neighbor length according to the aggregation gap distance, such as 2 mm in paper space. In Fig 10, steps 2-4 complete the continuous and conditional expansion, and the operation is stopped when no boundary triangle satisfies the expansion conditions. After performing step 4, we obtain two regions of the triangle set indicated by light red and light green, respectively. The building object associated with the triangle set can be assigned to one group.

**Fig 11:**
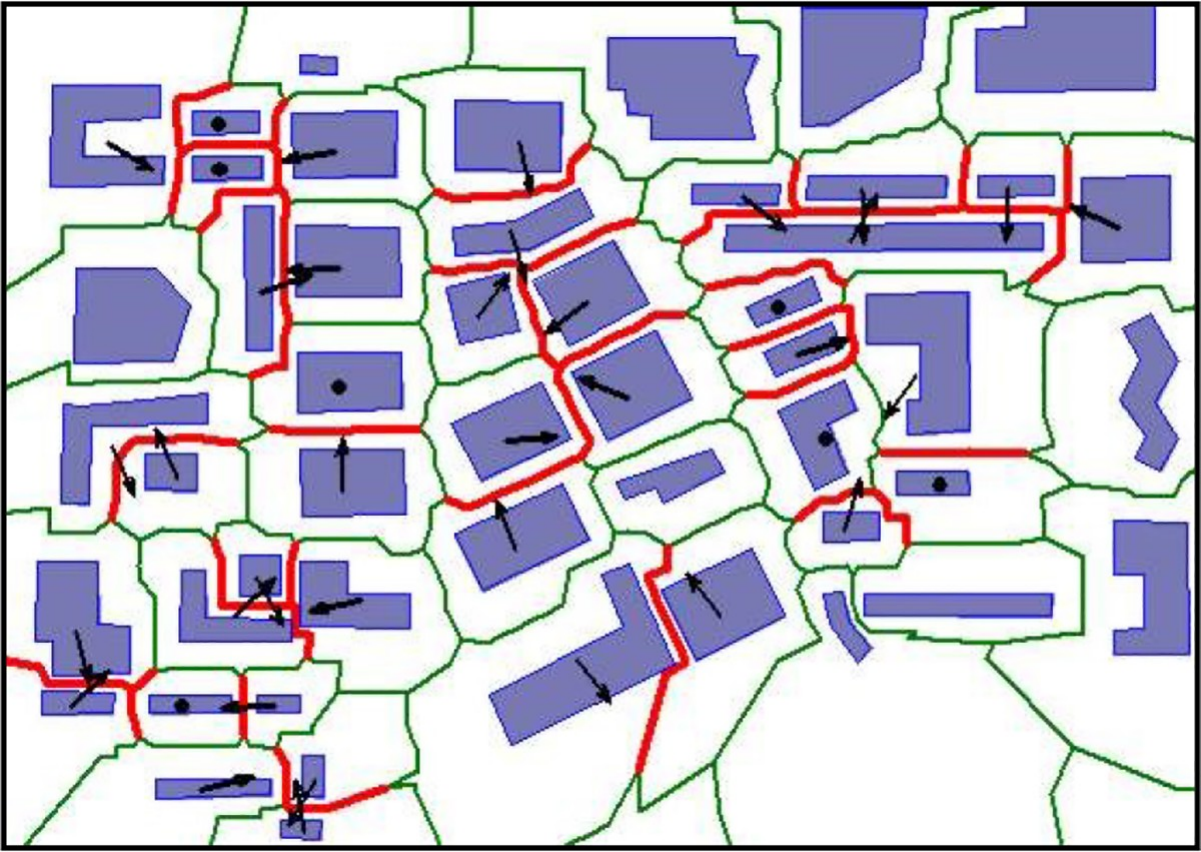
An illustration of the conflict skeletons, conflict OPs (Object Polygon) visualized as red lines, and the arrows represent the building movement direction.

Before step 5 in Fig 10, we observe that one building group is connected by a set of triangles locating in the gap area. The narrow region with a gap distance of less than tolerance *d* has been detected. For each set of triangles connecting gap area, we perform the operation *skeleton(r)* and obtain the skeleton central line between the neighboring buildings as shown in step 5. We define the skeleton using a gap distance of less than the tolerance *d* the conflict skeleton. An example of the detection of the conflict skeleton and conflict building is illustrated in Fig 11.

### 4.2 Displacement within one group

The building facing the conflict skeleton is called the conflict building. The neighboring closed conflict buildings move together before the aggregation as shown in step 6 of Fig 10. The displacement in the building cluster generalization is aimed at keeping built-up area balanced.

The analysis of the conflict buildings offers an answer to the question of which one will be displaced in later generalization. How far, and in which direction, the conflict building will move are to be determined next. The normal direction of the line of the conflict skeleton fitted by the least square method will be the moving direction, as indicated by the arrow symbol in Fig 11. When the conflict object has only one conflict skeleton, the moving direction is determined. Else, the integrated moving direction can be calculated by using the vector sum by the parallelogram rule. It is estimated that neighbor conflict attracts each conflict building with the same attraction force. One building will stay unchanged if it is attracted by neighbors from two opposite directions or is surrounded by conflict buildings (i.e., all skeletons related to one building are conflicted by each other). In a practical application, it can be considered that no one direction attraction is stronger than any other direction and that the object is fixed if the length of the sum vector is shorter than a threshold. The movement direction of the conflict object is illustrated in Fig 11 by a dark arrow symbol representing the displacement direction and a dark dot representing the fixed building.

In terms of the offset length of the displacement, it is assumed that the position accuracy is at least half the conflict distance, which means that the in-face movement and the meeting in one position of the conflict building are within the position accuracy. We draw extended lines that are parallel with the displacement direction, from each vertex of the conflict building and then compute the distance between the start vertex and the intersection point of the extended line and skeleton. The shortest one is set as the displacement offset length. It can be guaranteed that the movement does not result in the building crossing the envelope skeleton or its overlapping with other neighboring buildings. No other new conflicts are produced from this type of displacement; in displacement generalization research, this is essential.

However, generally speaking, after the above displacement, it is not guaranteed that two buildings will share exactly the same boundary seamlessly. Usually, small gap areas still exist. One possible solution to this issue is applying a rotation to it, even though it is difficult and complicated to decide the angle and range of rotation and solve the problem perfectly.

### 4.3 Aggregation of neighboring buildings

Steps 7 to 9 in Fig 10 illustrate the aggregation of neighboring buildings within one group. After the displacement of conflict building together, however, there still are small gap areas among the buildings [41]. It is necessary to construct an envelope polygon to cover all the buildings within one group. A simple method to achieve this is to use a basic operations buffer and overlap in common GIS functions. We first use an expansion buffer operation to exaggerate each building area, then use union overlap to obtain the coverage polygon, and finally perform a compression buffer to shrink back. The obtained result is an aggregated polygon. In our experiment, we apply the method based on the Delaunay triangulation again. Through the close connection with the Delaunay triangulation to extract the envelope polygon. This method has been described in the SDS model by Jones and Ware [25, 26].

### 4.4. Progressive generalization process

The group detection and detailed geometric operations in building data generalization is discussed above in Fig 10. The original data can be required from this website: https://pan.baidu.com/s/1Zr_H9cvdEvX3QzOhK3ASXwv (extracting code: jnxx). For the whole working process, it is necessary to organize the operators using some parameter control. Let us consider the case in which conflicting building object associate with each other. By moving and aggregating one conflict building into its neighbor, the conflict on the neighboring location may be also eliminated. The conflict among the parts can be solved by the displacement and aggregation of partly conflicted objects. Thus, we can use a progressive strategy to aggregate the conflict buildings. The progressive generalization process is described as follows.

We repeat the following steps until no conflict is found:

(1) Construct DTN triangulation, and find conflict skeleton and conflict building object based on FTDM model.
(2) Classify buildings into different groups according to the conflict skeleton connection.
(3) Scan the building group and use the method in Fig 10 to aggregate the closed neighboring buildings.
(4) Eliminate the remaining conflicts after the aggregation.

**Fig 12:**
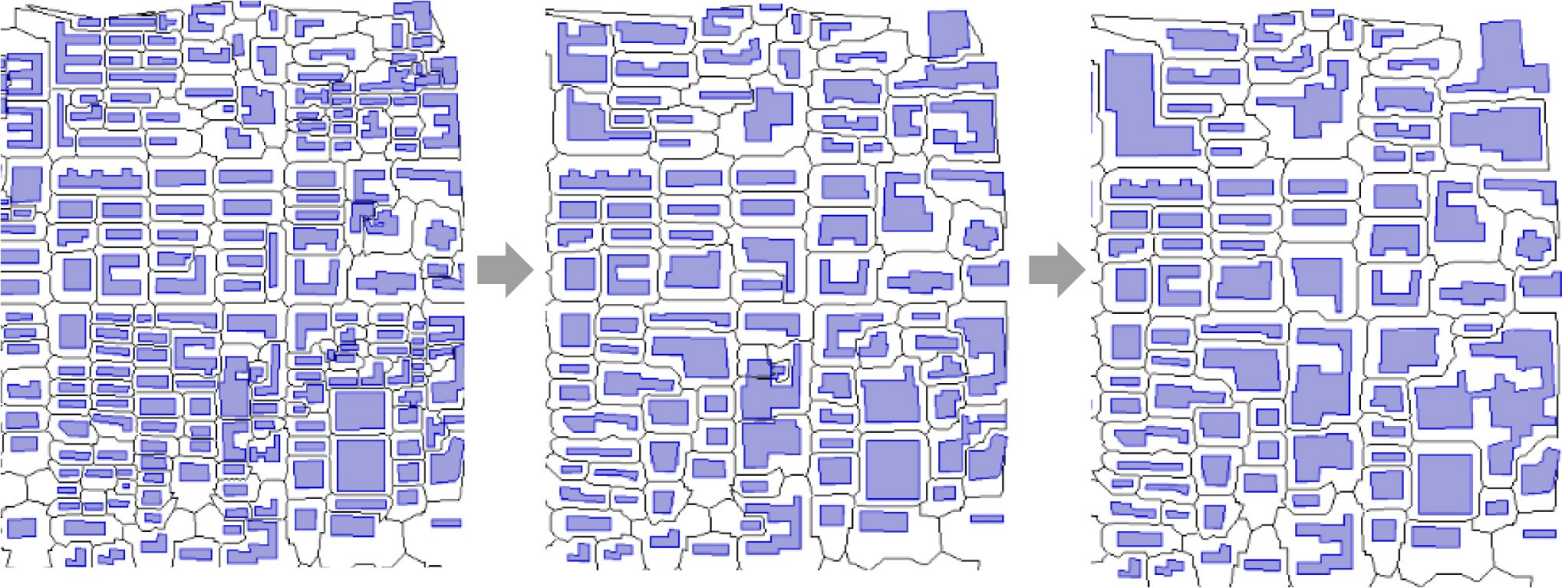
The progressive generalization of a building cluster based on the FTDM model.

Fig 12 presents some steps of the building cluster generalization and the generalized results as well as tessellation results. If the building is distributed in a common situation without a too-crowded distribution, a general appropriate outcome can be obtained using the above working process. As can be observed from the regions indicated by the red box in Fig 12, the original buildings are reasonably aggregated with the increase proximity distance, and the results are visually satisfactory. However, sometimes the location of the early aggregated building may produce a slight displacement as the skeleton is changed gradually throughout the process. In this method, the degree of generalization depends on the definition of the tolerant conflict distance.

### 4.5. Experiment discussion

**Fig 13:**
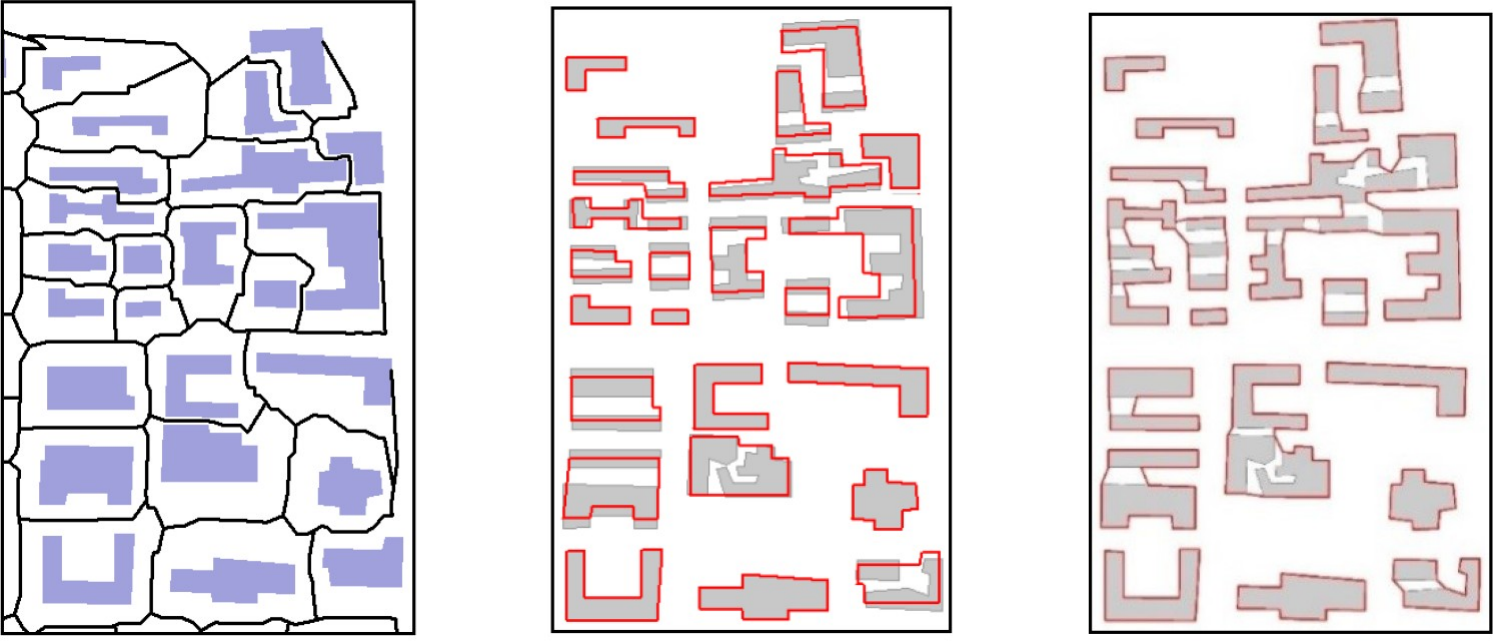
A comparison of building aggregation between the proposed method (B) and ArcGIS method (C).

Based on the FTDM model and formal operations *Expand(r)* and *Skeleton(r)*, we develop the method of building data generalization, which takes into consideration three operations in generalization, that is, grouping, displacement, and aggregation (the other operation of simplification of the building shape is not involved in this study). The advantage of this generalization is that the displacement before the aggregation maintain the balance of the built-up area as well as possible. The property of the remaining built-up area without a large change is an important requirement in map generalization [42, 43]. In this proposed method, the neighboring buildings move toward to each other and eliminate the gap area between them as well as possible. However, this cannot guarantee that two buildings share exactly the same boundary seamlessly. Small gap areas still exist, but the involved gap area reduces greatly. Some other aggregation methods, such as those used in [25, 20], aggregate the neighboring objects directly, thus resulting in a significant increase in the built-up area. We conduct an experiment of building aggregation based on the same data using the aggregation function in ArcGIS and our proposed method respectively. The comparison between the two methods can be made observed in Fig 13 and Table 1.

**Table 1:** The quantitative comparison between our proposed method and that of ArcGIS.

Method	Original area (m^2^)	Aggregated area (m^2^)	Change rate
ArcGIS method	37596.6	44137.7	17.4%
Proposed method	37596.6	39476.3	5.1%

The left side of in Fig 13 illustrates the aggregation result and the skeleton tessellation obtained using our method, the middle the aggregated result overlapping with original building data, and the right the same operation using the ArcGIS function. In Fig 13B, the buildings after aggregation using the proposed method are not inclined to adhere to the original buildings. However, the aggregated buildings using the ArcGIS method are inclined to adhere to the original buildings, as shown in Fig 13C. In ArcGIS ToolBox, we apply the command “Aggregate Polygons” the function of which is to aggregate the neighboring polygons. We set the tolerant gap distance *d* = 15 m for both the methods. From the comparison, we can observe that the aggregation performed using the ArcGIS function includes gap areas and greatly increases the built-up area. In Table 1, we find that the proposed method increases the built-up area by 5.1%, which is less than 17.4% for the ArcGIS aggregation method.

**Fig 14:**
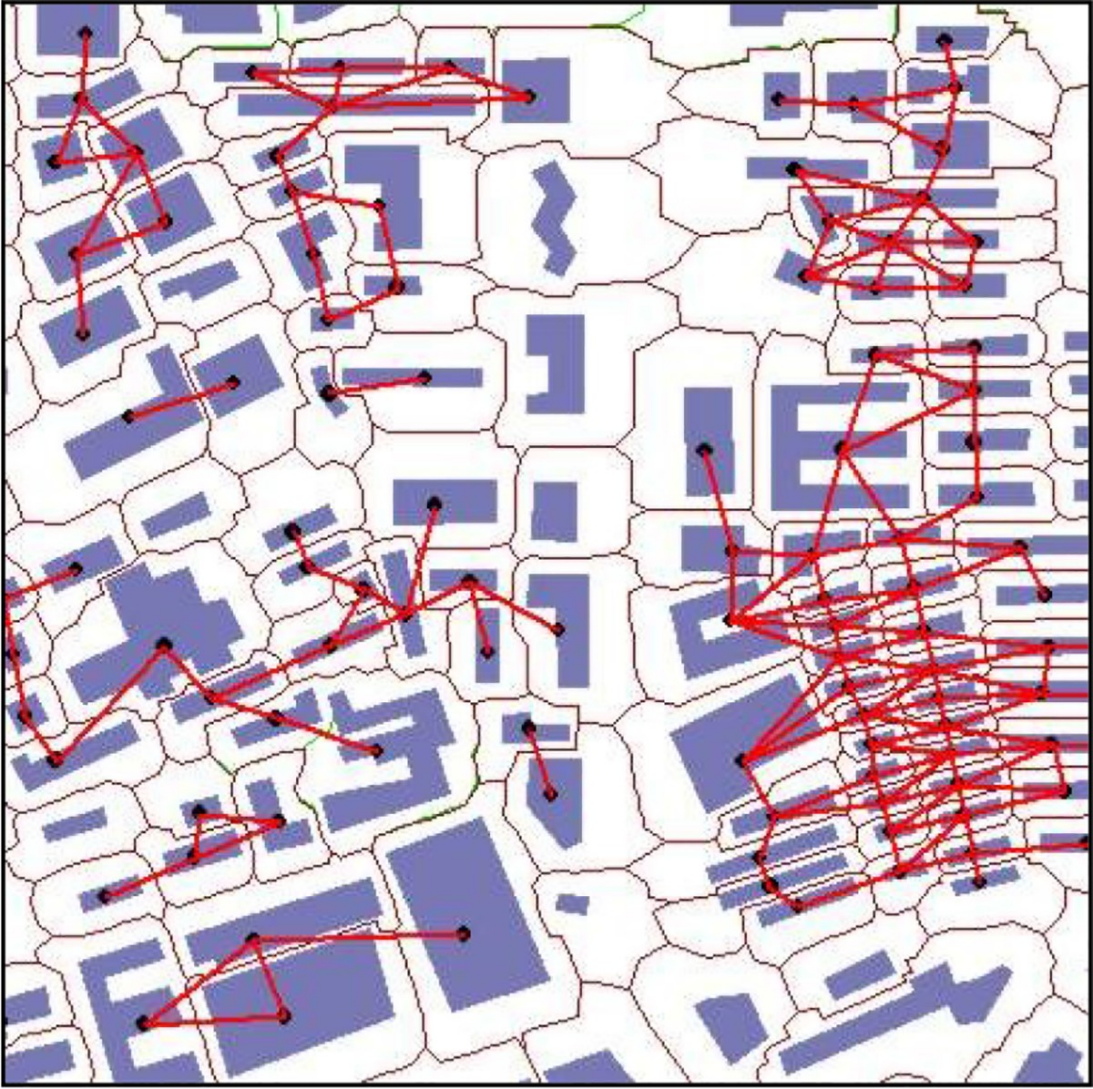
The network of a connective conflict building object.

The building generalization is aimed at resolving the issue of crowded building distribution in a small space. If the room competition and spatial conflict is serious in the absence of additional room for displacement, the proposed method then does not work effectively. For such a situation, the conflict skeletons account for a large rate, and the building group detection is required to take into consideration a greater number of aspects. One improvement strategy is to use the graph structure to analyze the building pattern by conflict object connection. A dual geometric construction and Delaunay triangulation can be reached by connecting the center points within the tessellated polygon. Accordingly, a number of conjoint networks can be produced by connecting the geometric central points of the conflicting building on the basis of the building partitioning model, as is presented in Fig 14. With the combination of other approaches, the future assignment is to discover building distribution patterns based on the proposed model of neighborhood representation.

## 5. Conclusion

In spatial data handling, the neighborhood relationship plays an important role in map generalization, co-location detection, and other applications. In addition to the buffering operation, the triangulation is also an efficient tool in neighborhood analysis. Delaunay triangulation, as a special geometric construction, is widely applied in map generalization for detecting neighboring regions or conflict regions. A large number of concrete applications of Delaunay triangulation in generalization algorithm design and data model development have been explored. However, the previous application models have not been formally represented. From the computation perspective, it is necessary to establish a formal model based on the Delaunay triangulation for neighborhood representation.

In this study, we build an FTDM data model based on the representation of the spatial field on the fundamentals of Delaunay triangulation, which combines the basic concept of a regular grid raster model and the advantage of the Delaunay triangulation on the spatial neighbor relationship representation. This paper presents the formal definition and operating algorithm of three types of operators, which correspond with the raster operation of a regular raster. The three operators play two roles: one takes a triangular vertex, edge, and area as the components of spatial objects of point, line, and region; the other takes the connection between the triangles as the components of the spatial neighbor relationship between spatial objects. This model can be used widely in the map generalization, detection of conflict in spatial data mining, merging of non-connected objects, and the representation of different spatial neighbor relationship classes.

To illustrate the application of the FTDM model, we examine the generalization of urban building clusters, which proves to be reasonable and effective. In this proposed method, we consider the building generalization through three steps of operations, i.e., grouping, displacement, and aggregation. In the decision-making stage, the grouping is used to detect the cluster pattern, which has to be preserved after abstraction. In the geometric operation stage, the displacement operation attempts to move buildings within a group together to avoid increasing the built-up area, and the aggregation operation attempts to combine the closed building group into one block. The algorithm complexity of the proposed method is O(nlogn), which satisfies the needs of general map generalization. The proposed FTDM model and the formal operator *Expand(r)* and *Skeleton(r)* can provide for conducting the three aforementioned operations. Through the triangle tessellation in the FTDM model and the neighborhood analysis by *Expand(r)*, we can detect the building group and conflict area and further, through *Skeleton(r)*, move closed buildings together. Using the FTDM-based generalization operations, the consistency of the overall distribution pattern of buildings can be well maintained. The relative position relationships of buildings are well considered and the neighborhood topological relationships of buildings will not be destroyed. However, in this method, it is difficult to establish one to one relationship of objects before and after building generalization and quantitatively evaluate the scale relationship change of each object due to the integrated processing of displacement and aggregation. Through the experiment comparison between our method and that of the ArcGIS function, the built-up area increment by our method is less than one third of that in the ArcGIS method (5.1% VS 17.4%). In addition to areal features, the FTDM model can also be applied for map generalization of point, line features, such as line simplification [33, 34], building displacement [29] and point cluster simplification [44]. As this study focuses on the establishment of FTDM model and the space is limited, the detailed applications in map generalization of point, line features will not be described in this paper.

However, some problems are still encountered while applying the proposed data model, such as that the original distribution pattern of the urban building cluster may be damaged and the position accuracy of some early aggregated buildings cannot be maintained as they are displaced in the whole process [28, 45]. In the future, the proposed method should be improved with respect to the distribution pattern and positional accuracy, especially in the remaining distribution pattern. In addition, in the process of building generalization, many aspects such as the geometrical, semantic characteristics and distribution pattern of buildings should be considered. A perfect generalization strategy based on the proposed data model should be further investigated.
